# Intensive Treatment for Upper Limb Lymphedema

**DOI:** 10.7759/cureus.18026

**Published:** 2021-09-16

**Authors:** Ana Carolina Pereira de Godoy, Maria de Fatima Guerreiro Godoy, Lívia Maria Pereira de Godoy, Henrique Jose Pereira de Godoy, Jose Maria Pereira de Godoy

**Affiliations:** 1 Pediatric Intensive Care, Hospital da Criança e Maternidade (HCM) Hospital de Base de Sao Jose do Rio Preto, Sao Jose do Rio Preto, BRA; 2 Pediatric Rehabilitation, Clínica Godoy, Sao Jose do Rio Preto, BRA; 3 Occupational Therapy, Clínica Godoy, São José do Rio Preto, BRA; 4 Dermatology, Instituto Lauro Souza de Lima-Bauru, São José do Rio Preto, BRA; 5 General Practice, Clínica Godoy, Sao Jose do Rio Preto, BRA; 6 General Surgery, Faculdade de Medicina de Sao Jose do Rio Preto (FAMERP) Clínica Godoy, São José do Rio Preto, BRA; 7 General Practice, Clínica Godoy, São José do Rio Preto, BRA; 8 Cardiology and Cardiovascular Surgery, Faculdade de Medicina de Sao Jose do Rio Preto, Sao Jose do Rio Preto, BRA; 9 Angiology and Vascular Surgery Service, Clínica Godoy, Sao Jose do Rio Preto, BRA

**Keywords:** rehabilitation, treatment, breast cancer, upper limbs, lymphedema

## Abstract

Introduction

Breast cancer-related lymphedema (BCRL) is a complication of treatment for breast cancer. The aim of the present study is to report a form of intensive treatment for BCRL.

Method

A crossover study was conducted involving the evaluation of the change in the volume of the upper limbs of 45 women with BCRL who underwent the intensive Godoy Method® (eight hours/day for five days). Volumetric analyses were performed before and after treatment and differences were analyzed using the paired t-test. Reductions in volume were found in all patients.

Results

The average reduction was 45.38%. The reduction was between 15% and 20% in 6.67% of the women (n = 3); 20% to 30% in 13.33% (n = 6); 30% to 40% in 20% (n = 9); 40% to 50% in 40% (n = 18); and more than 50% in 20% of the women (n = 9).

Conclusion

The intensive form of treatment for lymphedema is highly effective in a short period of time, with a 40% to 50% reduction in volume in five days, but requires specialized centers adapted to this form of therapy. This is an option for reference centers in the treatment of lymphedema and the formation of human resources.

## Introduction

Breast cancer-related lymphedema (BCRL) is a negative consequence of treatment for breast cancer with an incidence ranging from 24.8% to 90.4%. Several factors have been associated with lymphedema following conservative breast surgery, such as the body mass index (BMI), breast size, tumor size, tumor location, type of surgery and adjuvant therapy [1,2].

Based on the findings of two studies, evidence for the effectiveness of lymphovenous anastomosis in the prevention of the development of BCRL is low [3,4]. A study involving lymphoscintigraphy found that the restoration of the original lymphatic route to the axilla following nodal dissection was not uncommon and may prevent lymphedema. Regenerated lymphatic vessels contributed to the restoration of the original route or redirecting of the lymphatic route to other regional nodes [5].

A systematic review analyzing intervention studies involving resistance exercise for breast cancer survivors and the effect on BCRL found such heterogeneous evaluations that a complete meta-analysis on the status of lymphedema was impossible [6].

Another systematic review reports evidence that manual lymphatic drainage (MLD) in the early stages after breast cancer surgery may assist in preventing the progress to clinical lymphedema. MLD may also provide additional benefits regarding the reduction in volume in cases of mild lymphedema but does not provide any additional benefits when combined with complex decongestive therapy in cases of moderate to severe lymphedema [7]. A meta-analysis of 12 randomized clinical trials showed that MLD does not significantly reduce or prevent lymphedema in patients with BCRL [8,9].

In terms of treatment, a combination of therapies have been used for decades and involves manual lymphatic drainage, exercises, compression mechanisms and hygienic care [10,11]. In recent years, Godoy & Godoy developed novel concepts and techniques for the treatment of lymphedema, proposing clinical normalization or near normalization even in cases of elephantiasis [12]. Studies in the publication phase involving the evaluation of biopsies confirm clinical normalization. A pilot study shows that it is possible to reduce the volume of edema by approximately 50% through intensive treatment [13]. The aim of the present study is to report a form of intensive treatment for BCRL

## Materials and methods

Population and setting

The sample was composed of selected 45 women whether randomly. The chosen sample N calculations where the minimum number of patients could be 15 patients, with upper limb lymphedema. Mean age was 59.3 ± 10.2 years (range: 37 to 82 years). The evaluations and treatment were performed at the Clínica Godoy-Sao Jose do Rio Preto-Brazil.

Design

A prospective cohort study was conducted involving the evaluation of the change in the volume of the upper limb of 45 women with BCRL who underwent the intensive Godoy method® (eight hours/day for five days). Volumetric analyses were performed before and after treatment and differences were analyzed using the paired t-test.

Inclusion criterion

Patients submitted to breast cancer treatment who developed upper limb lymphedema.

Exclusion criteria

Primary lymphedema or lymphedema due to causes other than treatment for breast cancer and other causes of edema associated with lymphedema diagnosed at the initial clinical evaluation.

Statistical analysis

Descriptive statistical analysis of the data was performed and the paired t-test was used for the comparisons, considering a 5% alpha error.

Ethical considerations

This study received approval from the institutional review board of the São Jose do Rio Preto School of Medicine #773.336.

Development

The participants had a clinical diagnosis of lymphedema based on the clinical history and physical examination. Complementary volume measurement exams confirmed the volumetric diagnosis. Volumetry was performed using the water displacement method, circumference measurements and multi-segment, multi-frequency bioimpedance analysis (In Body S10). After agreement to participate in the study, the patients were submitted to intensive treatment with the Godoy Method®, which consisted of 20 minutes of cervical lymphatic therapy (approximately 30 gentle surface movements of 0.5 cm in the cervical region), six to eight hours of mechanical lymphatic therapy using the RAGodoy® device, which is an electromechanical device that performs 15 to 18 passive elbow flexion and extension movements per minute, one to two hours per day of manual lymphatic therapy adapted to the characteristics of the edema and a compression mechanism (hand-crafted sleeve made of grosgrain-non-elastic) fabric adjusted when necessary one to two times per day. On the first day, bandages were used for compression while the arm brace was being constructed. After the fifth day, volumetry was performed again. The data were entered into a table of the Excel program. The Stats Direct 3 program was used for the statistical analysis and the graph was created in Excel.

## Results

**Figure 1:**
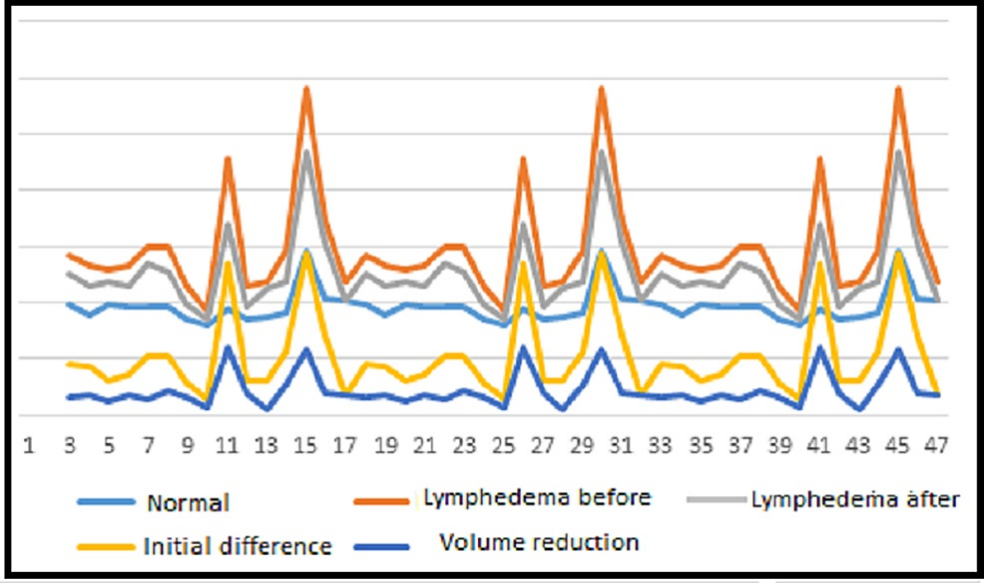
Difference and reduction of volume (ml) of normal upper limb and lymphedema upper limb before and after treatment.

Reductions in volume were found in all patients. The average reduction was 45.38% 95% CI = -457,23 to -388,36. The reduction was between 15 and 20% in 6.67% of the women (n = 3); 20 to 30% in 13.33% (n = 6); 30 to 40% in 20% (n = 9); 40 to 50% in 40% (n = 18); and more than 50% in 20% of the women (n = 9). Table 1 displays the data of the descriptive analysis of the normal upper limb and lymphedema upper limb before and after treatment as well as the difference in volume after treatment compared to before treatment. Significant differences were found between the normal upper limb and lymphedema upper limb (p = 0.0001, t-test) as well as between the initial and final volumetric analyses (p = 0.0001). Figure 1 shows the initial volumes of the normal upper limb and lymphedema upper limb before and after treatment as well as the reduction in volume of the lymphedema after treatment.

**Table 1:** Descriptive statistics of data before and after treatment and reduction in volume (ml) after treatment.

Variables	Normal	Before	After	Initial difference (ml)	Volume reduction (ml)	% reduction
Valid data	45	45	45	45	45	43
Mean	1922.2	2961.8	2538.1	1049.1	438.9	45.41
Standard deviation	296.39	977.13	708.70	746.92	305.84	18.83
Standard error of mean	44.44	145.92	105.90	111.60	45.85	2.78
Maximum	2912	5801	4645	2881	1175	100
Upper quartile	1960	2978	2673	1095	422	51.89
Median	1917	2639	23449	861	351	42.12
Lower quartile	1740	2355	2018	591	281	36.44
Interquartile range	222	625	657	506	138	15.46
Minimum	1579	1862	1715	284	100	16.07
Range	1338	3940	2934	2600	1078	83.90
Centile 95	2915	5800	4647	2883	1177	100
Centile 5	1577	1860	1713	282	98	16.07

## Discussion

The present study reports an important reduction of more than 45% in the volume of the upper limb with lymphedema following five days of intensive treatment using the Godoy Method®, changing the lives of these patients in only a short period of time. There are no studies in the literature reporting similar results. The authors initially evaluated combined therapies for longer periods of time, finding reductions of 58% in the first year, 82.2% in the second year and 97.02% in the third year. Another study with a weekly follow-up for an average of 12.3 months also reported a significant reduction [14]. Thus, the present study offers a novel treatment for lymphedema that accelerates the rehabilitation process.

The intensive therapy involves eight hours of treatment per day, which poses a set of challenges, but with an average reduction of 40 to 50% in the volume of the affected limb. The first challenge is the period of eight hours daily. Without six to eight hours of mechanical lymphatic therapy, it would be very difficult to achieve such results. Manual therapy is performed an average of one to two hours per day and cervical lymphatic therapy is performed 20 minutes per day. One option is to adapt the method to each treatment center. We suggest at least three times per week on alternate days for one hour and maintaining compression at least during the day until reaching normality in the reduction phase, at which point the maintenance phase begins [15].

The main compression mechanism we use consists of laced braces made with grosgrain fabric, which we have been perfecting over the years. Compression with either the braces or bandages should be performed 24 hours per day in this phase and maintained for an additional one to two weeks at night to achieve better results.

Cases in which additional harm occurs beyond that caused by treatment for cancer, such as infection and inadequate compression and massage techniques, which many healthcare providers erroneously denominate lymphatic drainage, pose greater challenges and lead to more hours of treatment in order to achieve a reduction in volume. Therefore, secondary harm caused after treatment for cancer is the main limiting factor that we have encountered, which suggests that healthcare providers should be well qualified and patients should be counseled in order to avoid the occurrence of further harm. In patients with mild lymphedema and without additional harm, average reductions in volume surpass 50% in the majority of cases [12]. Moreover, continued treatment can lead to the same volumetry as that found in the contralateral limb in two to four weeks.

After the complete reduction in volume, braces made with grosgrain fabric are the main form of treatment that we use. However, this can be alternated with elastic sleeves in this phase, which is an important and esthetically more pleasing option.

As lymphedema has no cure, it is important to use all surgical strategies to reduce its incidence in breast cancer survivors, such as the sentinel lymph node approach [16]. Immediate postoperative care is fundamental for the reduction in the occurrence of infection, hematomas and complications.

The articles cited in the introduction assist in the analysis of the postoperative options. The most attractive is the development of collateral circulation over time, which can avoid lymphedema or reduce edema in some cases. Therefore, every step should be taken to avoid additional harm, such as erysipelas, inadequate exercises without professional monitoring, inadequate lymphatic drainage, etc. In our personal experience, these are the factors that most exert a negative impact on treatment.

Immediate reconstruction surgery seems not to have any encouraging scientific evidence, but we believe that surgeons should continue performing it. Novel microsurgical techniques have made considerable advances and very satisfactory results may be found in patient who develop lymphedema.

Regarding manual lymphatic drainage, meta-analyses report some evidence in mild cases, but not in more advanced cases. I believe that several techniques employed can interfere with the results. However, in more advanced cases, a combination of therapies is necessary. Otherwise, a much larger number of sessions would be required to achieve good results. It is unlikely that patients have such time available and the cost would be very high.

Over the years, we have developed the RAGodoy® device for mechanical lymphatic drainage of the upper limbs, which assists considerably in both treatment and the maintenance of the results. Mechanical lymphatic drainage as monotherapy reduces the edema, but better results are achieved when combined with compression mechanisms [17-19]. Well-adjusted compression mechanisms are the most important in terms of the synergic effect in the reduction of edema and maintenance of the results.

Additional care is important, such as the control of obesity, pain, especially in women with lipedema, which are limiting factors and further reduce the quality of life of these patients [20-23]. The two main challenges in the treatment of BCRL are the normalization or near normalization of the edema and the maintenance of the results. Thus, every strategy to ensure adherence and facilitate treatment using simple but effective measures is fundamental. Counseling patients on the performance of activities of daily living while using compression devices is the best strategy for maintaining the results, but constant follow-up is necessary.

## Conclusions

The intensive form of treatment for lymphedema is highly effective in a short period of time, with a 40% to 50% reduction in volume in five days, but requires specialized centers adapted to this form of therapy. This is an option for reference centers in the treatment of lymphedema and the formation of human resources.

## References

[1] Abouelazayem M, Elkorety M, Monib S (2021). Breast lymphedema after conservative breast surgery: an up-to-date systematic review. Clin Breast Cancer.

[2] Gillespie TC, Sayegh HE, Brunelle CL, Daniell KM, Taghian AG (2018). Breast cancer-related lymphedema: risk factors, precautionary measures, and treatments. Gland Surg.

[3] Pereira de Godoy JM, Azoubel LM, Guerreiro de Godoy Mde F (2011). Evaluation of a clinical model of breast cancer-related lymphedema. Breast J.

[4] Markkula SP, Leung N, Allen VB, Furniss D (2019). Surgical interventions for the prevention or treatment of lymphoedema after breast cancer treatment. Cochrane Database Syst Rev.

[5] Suami H, Koelmeyer L, Mackie H, Boyages J (2018). Patterns of lymphatic drainage after axillary node dissection impact arm lymphoedema severity: a review of animal and clinical imaging studies. Surg Oncol.

[6] Hasenoehrl T, Keilani M, Palma S, Crevenna R (2020). Resistance exercise and breast cancer related lymphedema - a systematic review update. Disabil Rehabil.

[7] Thompson B, Gaitatzis K, Janse de Jonge X, Blackwell R, Koelmeyer LA (2021). Manual lymphatic drainage treatment for lymphedema: a systematic review of the literature. J Cancer Surviv.

[8] Liang M, Chen Q, Peng K (2020). Manual lymphatic drainage for lymphedema in patients after breast cancer surgery: a systematic review and meta-analysis of randomized controlled trials. Medicine.

[9] Sen EI, Arman S, Zure M, Yavuz H, Sindel D, Oral A (2021). Manual lymphatic drainage may not have an additional effect on the intensive phase of breast cancer-related lymphedema: a randomized controlled trial. Lymphat Res Biol.

[10] Kostanoglu A, Tarakcı E (2021). Physical therapy enhances functions and quality of life in older patients with breast cancer-related lymphedema: a prospective experimental study. Niger J Clin Pract.

[11] Huang YY, Toh PY, Hunt C, Lin JT, Kamyab R, Ponniah AK (2021). Breast cancer treatment-related arm lymphoedema and morbidity: a 6-year experience in an Australian tertiary breast centre. Asia Pac J Clin Oncol.

[12] Pereira de Godoy JM, Guerreiro Godoy MF, Barufi S, Pereira de Godoy HJ (2020). Intensive treatment of lower-limb lymphedema and variations in volume before and after: a follow-up. Cureus.

[13] Godoy JMP, Godoy HJP, Godoy MFG (2016). Transdisciplinary Approach to Rehabilitation of Breast Cancer-Related Lymphedema. Charleston (USA): Amazon.com.

[14] de Godoy JM, Godoy Mde F (2013). Evaluation of a new approach to the treatment of lymphedema resulting from breast cancer therapy. Eur J Intern Med.

[15] Godoy JMP, Lopes RP, Godoy LMP, Godoy MFG (2017). Pilot study on the association of different compression mechanisms to maintain the results of lymphedema treatment over one year. Ann Med Health Sci Res.

[16] Shin YD, Lee HM, Choi YJ (2021). Necessity of sentinel lymph node biopsy in ductal carcinoma in situ patients: a retrospective analysis. BMC Surg.

[17] Bordin NA, Guerreiro Godoy Mde F, Pereira de Godoy JM (2009). Mechanical lymphatic drainage in the treatment of arm lymphedema. Indian J Cancer.

[18] de Fátima Guerreiro Godoy M, Guimaraes TD, Oliani AH, de Godoy JM (2011). Association of Godoy & Godoy contention with mechanism with apparatus-assisted exercises in patients with arm lymphedema after breast cancer. Int J Gen Med.

[19] Godoy Mde F, Pereira MR, Oliani AH, de Godoy JM (2012). Synergic effect of compression therapy and controlled active exercises using a facilitating device in the treatment of arm lymphedema. Int J Med Sci.

[20] Pereira de Godoy JM, Pereira de Godoy LM, Guerreiro Godoy MF (2020). Prevalence of subclinical systemic lymphedema in patients following treatment for breast cancer and association with body mass index. Cureus.

[21] Guerreiro Godoy Mde F, Pereira de Godoy LM, Barufi S, de Godoy JM (2014). Pain in breast cancer treatment: aggravating factors and coping mechanisms. Int J Breast Cancer.

[22] Pereira de Godoy JM, da Silva SH, Guerreiro Godoy Mde F (2008). Interference of the surgical treatment of breast cancer on personal hygiene. Breast J.

[23] Godoy MFG, Godoy JMP, Braile DM (2008). Pilot study with myolymphokinetic activities in the treatment of lymphedema after breast cancer. Indian J Physiother Occup Ther.

